# Gastric adenocarcinoma with high‑level microsatellite instability: A case report

**DOI:** 10.3892/mco.2023.2612

**Published:** 2023-01-26

**Authors:** Alejandro Alfaro, Daniel Zanabria, Alfredo Aguilar, Sergio A. Jimenez-Solano, Alejandra Zevallos, Williams Fajardo

**Affiliations:** 1 Department of Pathology, Hospital Nacional Dos de Mayo, Lima 15003, Peru; 2 Department of Pathology, Oncosalud-AUNA, Lima 15036, Peru; 3 Basic and Translational Research Unit, Oncosalud-AUNA, Lima 15036, Peru; 4 Faculty of Natural Sciences and Mathematics, Universidad Nacional Federico Villarreal, Lima 15007, Peru; 5 School of Medicine, Universidad Privada San Juan Bautista, Lima 15067, Peru

**Keywords:** microsatellite instability, gastric cancer, anemia, MutS homolog 3

## Abstract

Gastric cancer (GC) ranks fifth on the list of the most common malignancies worldwide. In Peru, gastric neoplasms are considered the second leading cause of mortality among males. Among the molecular subgroups of GC, microsatellite instability presents a favorable prognosis due to its hypermutated phenotype, which activates immunosurveillance. The present study describes the case of a 75-year-old patient, who was admitted in the hospital with a history of upper gastrointestinal bleeding and recurrent hospital admission, due to severe anemia. The patient presented with pale skin, normal vital functions, slight swelling of the lower extremities, and abdominal distention and bloating upon a physical examination. An endoscopic examination revealed an infiltrating circular ulcerated lesion. The histopathological analysis identified a moderately differentiated intestinal-type adenocarcinoma with pathological stage T3N0M0. Tumor genomic profiling demonstrated alterations in 15 different genes with a tumor mutational burden of 28 mutations/Mb. Finally, the patient underwent a partial gastrectomy without pre-operative chemotherapy. After 4 days, the patient presented with post-operative complications for which he was re-operated on. The patient did not survive. To the best of our knowledge, in the present case, pernicious anemia was an early sign of GC and a gastroscopy had to be performed. Furthermore, MutS homolog 3 alterations probably conditioned the presence of multiple frame-shift mutations.

## Introduction

Gastric cancer (GC) is one of the most common neoplasms worldwide, affecting mainly males at a later age (1,2). In Peru, GC is considered the main cause of cancer-related mortality in the general population and the second most frequent type of cancer among males (3). Furthermore, one of the main risk factors for GC in patients of Peruvian descent is *Hellicobacter pylori* infection. A study performed at the National Institute of Neoplastic Diseases (INEN) revealed that up to 60% of patients with GC presented with *Hellicobacter pylori* infection (4).

By contrast, the incidence of GC increases considerably with age. Of note, the 30-day post-operative mortality rate is higher in patients who are >70 years of age following a gastrectomy (5). Likewise, other studies have demonstrated that patients with GC who are >65 years of age have a higher risk of mortality following surgical resection, including a gastrectomy (6,7).

Finally, the molecular subgroup of GC with microsatellite instability (MSI) represents <10% of GC cases. However, MSI is considered a favorable prognostic biomarker, since its hypermutation phenotype activates immunosurveillance, establishing this as a promising candidate for immunotherapy (8). The present study describes the case of an older patient with recurrent genomic mutations in a moderately differentiated gastric adenocarcinoma with a torpid evolution.

## Case report

A 75-year-old male patient from Huaral (Peru) visited the Emergency Department of the Dos de Mayo National Hospital (Lima, Peru) on September 15, 2020 with symptoms of severe anemia and melena. The patient had a history of tuberculosis, upper gastrointestinal bleeding and gonorrhea. Likewise, with a significant family history, his father was deceased due to prostate cancer and he also had two siblings who had died due to malignant neoplasia in the stomach. The patient had visited another hospital 18 months prior to this, due to the presence of peripheral edema associated with mild fatigue. He was diagnosed with severe anemia (hemoglobin, 7 mg/dl) and was prescribed with drinkable iron. After 8 months, the patient returned to another hospital for type I feces on the Bristol scale, mild constipation, mild fatigue and increased volume in the lower extremities, and was again diagnosed with severe anemia (hemoglobin, 8 mg/dl). Ferrous sulfate treatment was administered. Following treatment, the patient reported that no improvement and continued to have intermittent dark stools.

Upon a physical examination, the patient exhibited pale skin, normal vital functions, slight swelling of the lower limbs, and abdominal distention and bloating. Laboratory tests revealed a hemoglobin level lower than normal (4 g/dl) and a normal biochemical profile.

**Figure 1:**
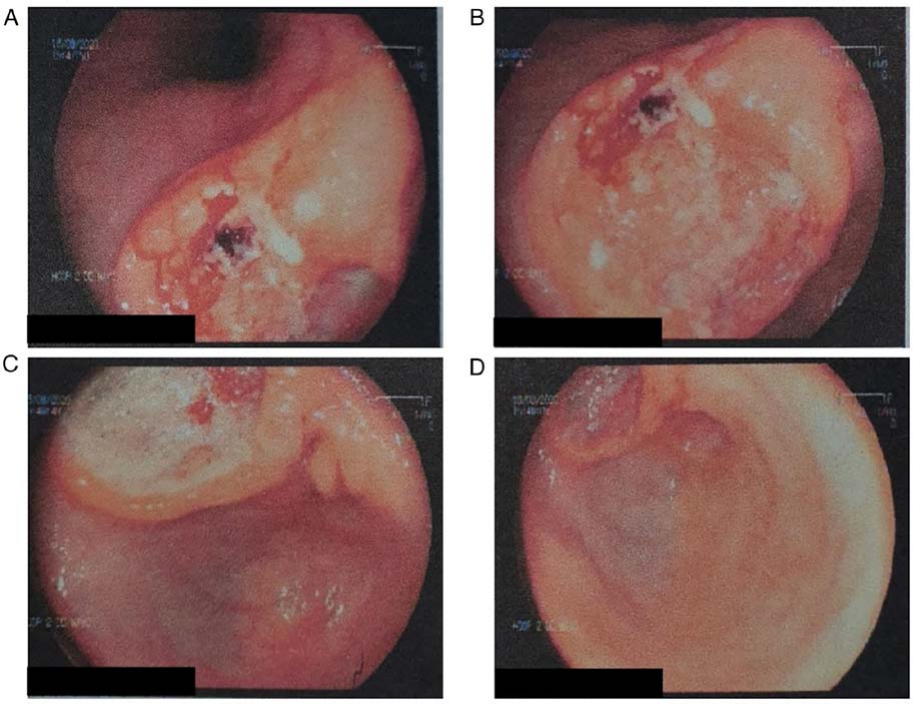
Upper gastrointestinal endoscopy. (A and B) Circular ulcerated mass of 5x3 cm located in the region of the (C and D) distal antrum and pylorus. The black rectangular boxes on the bottom left corner of each image have bene added for purposes of anonymity, namely to cover the patient's name.

The endoscopic analysis demonstrated a large circular ulcerated lesion, measuring 5x3 cm in size, which was detected in the lesser curvature of the antrum. The observable tumor border was irregular with edges that do not appear smooth, with encompassing fibrin deposition (Fig. 1). Furthermore, following the clinical practice guidelines of the Dos de Mayo Hospital, a biopsy of the stomach lining proband was obtained and this was found to be positive for *Helicobacter pylori*. Based on these findings, the patient underwent an open subtotal gastrectomy. The patient was treated according to the American College of Surgeons National Surgical Quality Improvement Program/American Geriatrics Society Best Practices guidelines (9).

**Figure 2:**
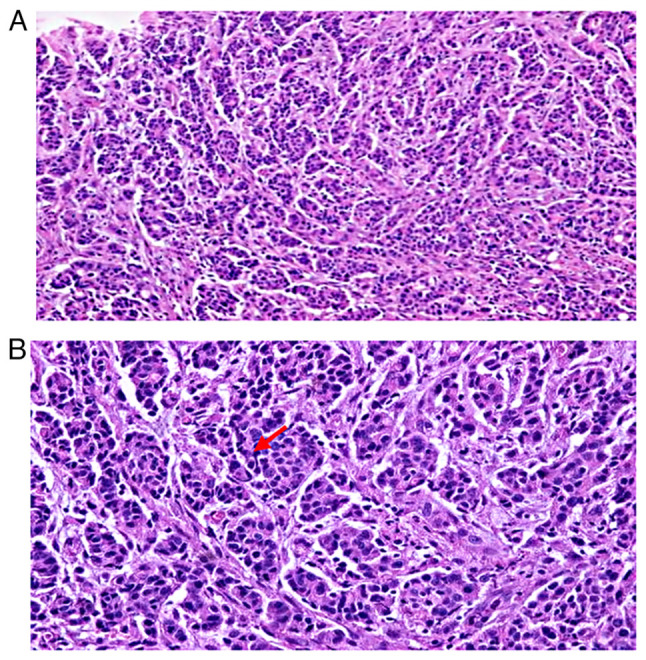
Histopathological analysis. Moderately differentiated tubular adenocarcinoma. (A) H&E, x10 magnification, (B) H&E, x40 magnification. The red arrow indicates moderately differentiated tubular structures.

Hematoxylin and eosin (H&E) staining was conducted on formalin-fixed and paraffin-embedded tumor tissue as described by Feldman and Wolfe (10). The stained tissues were cut into 5-mm-thick slices, dewaxed, mounted with neutral balsam and then imaged using a Leika DM100 microscope (Leica Microsystems, GmbH). This test revealed a moderately differentiated intestinal-type adenocarcinoma according to the Lauren classification (Fig. 2). Venous vascular invasion and surgical margins free of tumor involvement were observed. A total of 18 lymph nodes were examined with no evidence of neoplasia. According to the clinicopathological assessment by a pathologist (AAl), the pathological stage was defined as T3N0M0, and the clinical stage as IIB.

After signing a written informed consent, the patient was enrolled in a GC observational study (unpublished data); therefore, a genomic analysis was performed with targeted NGS using the platform FoundationOne CDx^®^ (Foundation Medicine, Inc.) as previously described by Frampton *et al* (11). Since the observational study was multicentric, the Via Libre Ethics Committee that is duly registered and accredited by the National Institute of Health in Peru was addressed. The genomic profile revealed a high microsatellite instability with a tumor mutational burden (TMB) of 28 mutations/Mb. Alterations were observed in 15 different genes [F-box and WD repeat domain containing 7 gene (*FBXW7*), T-rich interactive domain-containing protein 1A gene (*ARID1A*), *KRAS*, ring finger protein 43 gene (*RNF43*), ATR serine/threonine kinase gene (*ATR*), bromodomain-containing protein 4 gene (*BRD4*), caspase 8 gene (*CASP8*), cyclic adenosine monophosphate response element binding protein binding protein (*CREBBP*), folliculin gene (*FLCN*), Janus kinase gene (*JAK1*), *MAP3K1*, histone-lysine N-methyltransferase 2D gene (*MLL2*), MutS homolog 3 (*MSH3*), *SMAD4, SOX9*], 14 of which are related to frameshift mutations and one related to an amino acid substitution (*KRAS*) (Table I).

Finally, 4 days after the subtotal gastrectomy, the patient presented with post-operative complications and underwent two additional surgeries. The first surgery was performed due to an early small bowel obstruction caused by fibrous bands of tissue in the abdomen, forming after surgical procedures. The second surgery was performed due to wound dehiscence and stump infection. A peritoneal dissemination was produced by an anastomotic leakage, which led to an abdominal sepsis. After the second surgery, the patient developed septic shock and a multisystemic organ failure ultimately, leading to mortality.

## Discussion

Overall, >6 and <24% of patients with gastroesophageal cancer present with MSI (12-15), with an increase trend towards 48% in adults >40 years of age (16). Microsatellites are short and repetitive DNA sequences that are abundant in the human genome, particularly in non-coding DNA regions (17,18). Deficiencies in the mismatch repair (MMR) mechanism are caused by germline or sporadic mutations, which may result in nucleotide insertion or deletion in microsatellite regions during DNA replication. This phenomenon is known as MSI (18,19).

Individuals with mutations in the protein complexes that cause MMR have a high predisposition to develop neoplasms (20). In the patient treated in the present study, it was observed that *MSH3* presented alterations. *MSH3* is a member of the MMR system (21). Several studies have demonstrated its importance as a tumor suppressor gene (20-23). In the present study, the patient presented several frameshift mutations that may be related to the *MSH3* mutation. To date, studies have revealed that *MSH3* deficiencies are related to changes in the reading frame of microsatellite regions and may contribute to tumorigenesis (26,27). In total, >2 and <7.7% of patients with GC have mutations in *MSH3* (28,29). Similarly, the *MSH3* deletion has been found to be associated with increased chromosomal instability in p53-deficient tumors (25).

Within the classification of GC, the MSI-high (MSI-H) subtype predominates in elderly patients (>65 years of age), and is related to the intestinal type of the Lauren classification, being usually located in the distal part of the stomach, particularly the pylorus and gastric body, and presenting with a better prognosis (28-30). In this sense, MSI-H tumors are characterized by high levels of CD8^+^ T cell infiltration, thus being good candidates for immunotherapeutic treatments (33,34).

Several studies have revealed that TMB may be a promising predictive biomarker for immunotherapy in various types of cancer (35-40). To date, it has been revealed in the literature that patients with advanced-stage GC with a high TMB have a better clinical response to immunotherapy and demonstrate an improved overall survival (41). However, it is unclear whether TMB has clinical relevance for patients with advanced or metastatic GC (38,42,43). In addition, TMB usually is determined by a whole genome sequencing, thus limiting its clinical use, due to its increased cost and long turnaround times. In the case presented herein, the FoundationOne CDx panel was used to obtain the molecular profiling of the patient. Recently, two studies have demonstrated the feasibility and utility of this panel on clinical application with Japanese cohorts. The panel presented an assay success rate of 97,3% and physicians were able to give targeted therapy ≤14% of their population sample (44,45). Personalized medicine has not yet been implemented in routine oncological practice in Peru. However, there is an urgent need for the improvement of cancer diagnosis and prognosis, since this type of technology not only permits the provision of an efficient therapeutic regimen to all patients, but also increases drug accessibility to regional healthcare establishments (46).

By contrast, in the case in the present study, severe anemia was diagnosed. The study by Medrano-Guzmán *et al* (47) revealed that ≤2/3 of patients with GC had anemia as part of the initial symptoms. Furthermore, anemia was an adverse prognostic factor for survival (OR, 3.62; P<0.001; CI, 1.4-13.8) (47). Another study involving cancer patients revealed that anemia was a factor associated with mortality (HR, 3.04; P=0.002; CI:1.51-6.09), increasing the risk of mortality by ≤3-fold (P=0.008; CI, 1.35-7.05) (48). Likewise, anemia contributes to a hypoxic state of the tumor, which promotes angiogenesis; consequently, the attenuation or elimination of anemia improves survival and response to treatment (49). Similarly, *Helicobacter pylori* infection is a critical factor to consider for patients, as this has also been shown to be associated with MMR deficiencies and the presence of MSI (50,51). The study by Machado *et al* (52) demonstrated *in vitro* and *in vivo* that *Helicobacter pylori* infection reduced MMR activity, thus rendering gastric epithelial cells vulnerable to genetic instability, further contributing to gastric carcinogenesis in *Helicobacter pylori*-infected individuals.

The evolution of surgical technology has made habitual for physicians to perform surgical treatments in gastrointestinal cancer patients with advanced age. However, geriatric patients tend to present a compromised health state due to several factors, including comorbidities, frailty, geriatric syndromes, infections and transfusions, which increase their risk of post-operative complications and death (53). Likewise, it is important to highlight that patients with malnutrition present a higher risk of post-surgical complications including infections and other events (54-56). In the present case report, the patient died due to post-operative complications; a previous meta-analysis by Xue *et al* (57) demonstrated that the incidence of post-operative complications in geriatric patients with gastrointestinal cancer was between 24 to 76.3%. Early post-operative small bowel obstruction (ESBO) is present in ~1 to 12% of abdominal operations (58-61). Of note, the study by Nakamura *et al* (62) revealed that 10% of patients who underwent open surgery developed ESBO; thus, this type of surgery represents an independent risk factor for the develoment of ESBO (odds ratio, 5.621; P*=*0.015). By contrast, the literature reports that malnutrition and especially hypoalbuminemia are associated with poor healing processes, decreased collagen synthesis in surgical wounds, and decreased immune response. These factors determine the higher prevalence of surgical site infections and intestinal anastomosis leakage (63-65). Additionally, it should be noted that low hemoglobin counts, and hypoalbuminemia are common findings in patients undergoing surgery in public hospitals in Peru, since a number of these patients present chronic malnutrition (66-68).

Additionally, the barriers for the management of cancer patients in Peru should be highlighted. In the present case report, the patient was referred from other hospitals of primary care. The first symptom of the patient was a persistent anemia which lead to a misdiagnosis by previous physicians, contributing to the health deterioration of the patient. However, in the public sector there are delays not just in the counter-referral of patients to hospitals of more complexity, but also in the access to specialized cancer care, once a patient is referred to the new hospital (69). Literature reports that anemia is a common symptom in patients with GC (70). In that regard, it is important to develop and strengthen GC screening programs at the primary care centers.

Lastly, high-level microsatellite instability has been widely associated in the literature with better prognosis in GC patients and its improvement with immunotherapy has been described (18,30,71). The case described in the present study highlights the importance of carefulness concerning the treatment and management of geriatric patients with GC, particularly when it is necessary to perform radical treatments, such as gastrectomy. The probabilities of post-operative complications were also underlined, particularly in the Peruvian population, where patients generally present with malnutrition.

## Figures and Tables

**Table I:** Patient microsatellite instability analysis.

No.	Gene	Alteration	VAF (%)
1	FBXW7	C46fs*14	17.4
2	ARID1A	Y551fs*68	18.6
3	KRAS	G13D	17.3
4	RNF43	G659fs*41	39.6
5	ATR	I774fs*5	17.8
6	BRD4	P475fs*109	18.1
7	CASP8	K490fs*73	17.3
8	CREBBP	I1084fs*15	18.3
9	FLCN	H429fs*39	20.5
10	JAK1	P430fs*2	18.4
11	MAP3K1	L920fs*10	19.1
12	MLL2	G1235fs*95	16.7
13	MSH3	K383fs*32	30.1
14	SMAD4	S32fs*1	16.9
15	SOX9	P350fs*33, R264fs*32	25.1, 23.5

VAF, variant allele frequency.

## Data Availability

The data from the patient's molecular profile presented in the presesnt case report are available from the Figshare platform (https://doi.org/10.6084/m9.figshare.20522820).
